# Supplementary material for: Modeling glioblastoma heterogeneity as a dynamic network of cell states
Source: Mol Syst Biol. 2021 Sep 16;17(9):e10105. doi: 10.15252/msb.202010105 (PMC8444284; doi:10.15252/msb.202010105)
Supplement: Supplementary file 6 — Source Data for Figure 5 [file MSB-17-e10105-s004.zip › Figure5A_sourcedata/GSEA_3017/hallmarks_stateE.GseaPreranked.1621934677158/HALLMARK_MTORC1_SIGNALING.html]

Details for gene set HALLMARK\_MTORC1\_SIGNALING[GSEA]

|  || Dataset | state63017 |
| Phenotype | NoPhenotypeAvailable |
| Upregulated in class | na\_pos |
| GeneSet | HALLMARK\_MTORC1\_SIGNALING |
| Enrichment Score (ES) | 0.16725454 |
| Normalized Enrichment Score (NES) | 0.9004481 |
| Nominal p-value | 0.60096157 |
| FDR q-value | 0.59710115 |
| FWER p-Value | 0.882 |
Table: GSEA Results Summary

  

Fig 1: Enrichment plot: HALLMARK\_MTORC1\_SIGNALING      
 Profile of the Running ES Score & Positions of GeneSet Members on the Rank Ordered List

  

| PROBE | GENE SYMBOL | GENE\_TITLE | RANK IN GENE LIST | RANK METRIC SCORE | RUNNING ES | CORE ENRICHMENT || 1 | IGFBP5 |  |  | 4 | 1.015 | 0.0690 | Yes |
| 2 | CYP51A1 |  |  | 30 | 0.637 | 0.0878 | Yes |
| 3 | HMGCS1 |  |  | 37 | 0.593 | 0.1241 | Yes |
| 4 | HSPA5 |  |  | 73 | 0.449 | 0.1184 | Yes |
| 5 | SYTL2 |  |  | 82 | 0.437 | 0.1413 | Yes |
| 6 | VLDLR |  |  | 87 | 0.419 | 0.1673 | Yes |
| 7 | SQLE |  |  | 168 | 0.319 | 0.1030 | No |
| 8 | FADS2 |  |  | 181 | 0.310 | 0.1123 | No |
| 9 | ACSL3 |  |  | 207 | 0.291 | 0.1061 | No |
| 10 | IDH1 |  |  | 218 | 0.284 | 0.1157 | No |
| 11 | HMGCR |  |  | 251 | 0.261 | 0.0996 | No |
| 12 | INSIG1 |  |  | 252 | 0.261 | 0.1185 | No |
| 13 | TUBG1 |  |  | 323 | -0.263 | 0.0611 | No |
| 14 | HSPD1 |  |  | 329 | -0.264 | 0.0747 | No |
| 15 | GSR |  |  | 349 | -0.268 | 0.0734 | No |
| 16 | CDC25A |  |  | 368 | -0.273 | 0.0735 | No |
| 17 | PPA1 |  |  | 420 | -0.289 | 0.0388 | No |
| 18 | MCM4 |  |  | 457 | -0.301 | 0.0213 | No |
| 19 | UCHL5 |  |  | 487 | -0.310 | 0.0120 | No |
| 20 | NUP205 |  |  | 495 | -0.313 | 0.0270 | No |
| 21 | PIK3R3 |  |  | 508 | -0.317 | 0.0368 | No |
| 22 | CACYBP |  |  | 589 | -0.352 | -0.0251 | No |
| 23 | DDX39A |  |  | 590 | -0.353 | 0.0004 | No |
| 24 | MTHFD2 |  |  | 625 | -0.372 | -0.0098 | No |
| 25 | PSAT1 |  |  | 720 | -0.448 | -0.0800 | No |
| 26 | CCNF |  |  | 732 | -0.460 | -0.0587 | No |
| 27 | PLK1 |  |  | 809 | -0.586 | -0.0993 | No |
| 28 | AURKA |  |  | 824 | -0.623 | -0.0696 | No |
| 29 | BUB1 |  |  | 837 | -0.653 | -0.0354 | No |
| 30 | DHFR |  |  | 851 | -0.701 | 0.0011 | No |
| 31 | RRM2 |  |  | 940 | -1.403 | 0.0066 | No |
Table: GSEA details [plain text format]

  

Fig 2: HALLMARK\_MTORC1\_SIGNALING: Random ES distribution      
 Gene set null distribution of ES for **HALLMARK\_MTORC1\_SIGNALING**
